# Runoff Estimation of Jiulong River Based on Acoustic Doppler Current Profiler Online Monitoring Data and Its Implication for Pollutant Flux Estimation

**DOI:** 10.3390/ijerph192316363

**Published:** 2022-12-06

**Authors:** Zhi Zeng, Yufang Wu, Zhijie Chen, Quanjia Huang, Yinghui Wang, Yang Luo

**Affiliations:** 1 Third Institute of Oceanography, Ministry of Nature Resources, Xiamen 361005, China; 2 Xiamen Environmental Monitoring Station, Xiamen 361021, China; 3 College of Environmental Science and Engineering, Ocean University of China, Qingdao 266100, China

**Keywords:** Jiulong River Estuary, ADCP navigation observation, flow volume

## Abstract

The runoff of the Jiulong River (JLR) is a key parameter that affects the estimation of pollutant flux into Xiamen Bay (XMB). The precise runoff estimation of the JLR can be used to determine the accuracy of the pollutant flux estimation flowing into XMB. In this study, to analyze the hydrological dynamic characteristics and identify the correlation between fixed-site real-time ocean current observations and cross-sectional navigation flow observations, we conducted six navigation observations on two cross-sections of the JLR estuary during the spring tide and neap tide in the normal season, wet season, and dry season in 2020. Simultaneously, we measured hydrological observation data by a fixed-site buoy located in the JLR estuary and collected runoff data that were measured upstream of the JLR. The results showed that the average correlation coefficient between the average velocity of the fixed-point buoy and average velocity of the section was more than 0.90, higher than expected, the minimum average deviation was 4%, and the minimum sample standard error was 5.7%, which was a good result. In this study, we constructed a model for estimating the runoff of the JLR into the sea. The findings demonstrated that Acoustic Doppler Current Profiler (ADCP) online monitoring data were useful to estimate runoff of the JLR with high accuracy, could promote the accuracy of estimated pollutant flux of the JLR’s discharge into XMB, and could provide more scientific and reliable basic data for future load flux estimation research.

## 1. Introduction

The environmental pollution of estuaries and bays is an important research topic regarding the interaction between land and sea and is a key issue in the study of global environmental change [1]. In recent decades, with increasing human activities and the rapid development of social economy in coastal land areas, a large amount of pollutants has been discharged into coastal waters, resulting in an imbalance of the nutrient ratio in coastal waters, frequent red tide disasters, ecosystem degradation, and decline of ecosystem service functions [2]. These issues directly affect the overall, coordinated, and sustainable development of the social economy in coastal areas [3]. To improve the quality of the water environment in the coastal waters, the world’s major marine countries successively implemented a total pollutant control system with the goal of improving the quality of the marine water environment to alleviate the gradual deterioration of the national marine environment [4].

Total pollutant control, also known as total pollutant discharge control, total pollution load control, or total pollutant loss control, is a method of pollution control implemented by controlling the amount of pollutants generated by pollution sources within a certain space within an allowable environmental limit, in the comprehensive economic, technological, and social conditions during a specific period of time [5]. This concept was first proposed by Japan and the United States and was introduced in China in the early 1970s. It was originally used for surface water environmental management. During the “Twelfth Five-Year Plan” period, the concept was introduced into the marine field and was proposed for the “control of total pollutants entering the sea” Coastal cities have conducted pilot projects to establish the total amount of pollutants entering the sea based on the environmental capacity of their gulfs [6]. Although certain results have been achieved to date, they have also exposed problems, most of which are concentrated in the lack of accuracy in the response relationship between the current Gulf water quality and pollution source emissions. It is necessary to effectively evaluate the implementation effect of the total amount of pollutants entering the sea.

Total monitoring is a systematic method that integrates supervision and management, total monitoring, and quantitative evaluation. It has two main parts: water environment quality monitoring and flux monitoring. Conducting research on the online monitoring method of flux into the sea, through real-time monitoring of pollutant discharge and pollutant flux in various regions, this method provides a scientific basis for determining the total amount of pollutant flux in the coastal waters of China. Total monitoring can be used to understand the impact of rivers and other land-sourced pollutants on the coast. The degree of influence of the sea area provides strong technical support for the government in the management of marine environmental protection [7].

The two methods used to calculate pollutant flux are the direct estimation method, based on statistical analysis, and the indirect estimation method, based on numerical simulation. The traditional method calculates the net discharge flow of the exit gate using the monthly average flow of the upstream control station by the corresponding pollutant monitoring concentration to calculate the pollutant flux from the river network into the sea. This statistical method can be used to estimate and analyze the pollutant flux’s changing law and influencing factors. The numerical simulation method includes gridding the study area, simulating physical and biological processes by establishing models, and using the dynamic change process of flow at the estuary. This method uses the water mass obtained from the simulation results of the model to calculate the continuous flux of dynamic changes in real time [8].

The runoff in most estuaries is significantly affected by tides, and the pollutant concentration at the entrance also oscillates back and forth at the estuary with the tide, resulting in obvious changes in pollutant concentration and irregularities. The simple statistical method, of multiplying the net discharge and pollutant concentration, is used to estimate the flux of pollutants into the sea. This method depends on the frequency of water quality monitoring and the frequency of hydrological observations at the estuary.

Foster et al. (2000) [9] observed and measured organic matter, such as polychlorinated biphenyl (PCBs) and polycyclic aromatic hydrocarbons, in Chesapeake Bay in the United States over a one-year time span and multiplied this value with the annual runoff to calculate annual flux. They concluded that high-concentration emissions in urban areas were the main cause of serious organic pollutants in Chesapeake Bay. Webb et al. (1997) [10] used statistical methods to test the fluxes of a variety of different substances and established a regression relationship between sampling frequency and sampling method with accuracy and precision to obtain the maximum flux of different substances, in order to get the best estimation method. Liu Xincheng et al. (2002) [11] used years of measured flow and carbon, nitrogen, phosphorous, and silicon data from the Datong section of the Yangtze River to multiply the monthly average flow and average concentration data of this section of the river to obtain the monthly average flux of these four elements. The fluxes of the four elements in the flood season accounted for more than 60% of their total annual fluxes, which revealed the characteristics of seasonal changes in the high flood season and the low dry season. Shen Zhiliang (2006) [12] set up a total of 60 stations along 20 sections of the Yangtze River from Panzhihua to the mouth of the Jinsha River in the dry season in 1997 and the wet season in 1998 to measure the various forms of nitrogen in the samples. According to the monthly average flow at each section, the transportation and output flux of inorganic nitrogen in the Yangtze River Basin during the dry and wet periods were calculated. Nitrate nitrogen accounted for the majority of the flux, including the output and transportation of various forms of dissolved nitrogen, which accounted for the majority of inorganic nitrogen. The study concluded that the output flux of various forms of nitrogen in the Yangtze River estuary was controlled mainly by runoff and was closely related to human activities.

Sun Huiguo et al. (2006) [13] estimated the carbon flux in a catastrophic flood event on the Xijiang River, the main stream of the Pearl River, in June, 2005, and concluded that the annual fluxes of dissolved inorganic carbon (DIC), dissolved organic carbon (DOC), and particulate organic carbon (POC) during the flood period were 1.52 × 10^6^, 0.24 × 10^6^, and 0.54 × 10^6^·g km^−2^·a^−1^, respectively, which accounted for 14.9%, 24.8%, and 44.9% of the annual carbon flux, respectively. The carbon output during the flood period had a non-negligible contribution to the total annual carbon output of the river. Dong Dehua et al. (1993) [14] conducted a macroanalysis of the emission rules and composition of pollution sources in the Pearl River Basin, combined with the impact of runoff on the concentration of pollutants entering the sea, and verified that the eight major entrances of the Pearl River were dry and abundant. The annual flux in the three periods of the water period was analyzed to provide opinions on the sampling site, time, and frequency of the water quality observation of the Pearl River Estuary into the sea. Ni et al. (2008) [15] used sampling at the eight major entrances of the Pearl River Estuary from March, 2005, to February, 2006, during the neap tide and quantitatively measured total organic carbon (TOC) and suspended particulate matter (SPM), calculated the runoff according to the diversion ratio of each entrance, and then used statistics. The method estimated the flux of organic carbon and suspended particulate matter from the river network into the sea.

This previous research showed that the calculation of pollutant flux using traditional statistical analysis methods requires long-term and continuously measured data, the task load and economic cost are significant, and the accuracy and precision of the results obtained are not ideal. The forecasting ability is also limited. Due to the low frequency of routine water quality monitoring at the gate of the Pearl River, the frequency ranges from once in March to once in January, which is similar to European and American countries, and most of the sampling timing is concentrated at low tide. The limitation of frequency means it has been difficult to study the dynamic process of pollutants flowing back and forth under the influence of tides at the entrance through field sampling. Therefore, the pollutant fluxes calculated by traditional statistical methods in previous studies were merely observational. The instantaneous flux at a given time did not represent the true flux into the sea during the entire calculation period.

Hu Zhanming et al. (2018) [16] took the Liaohe online monitoring test base as a case study and used its 2016 high-water period online continuous monitoring data to monitor the representative sites, representative layers, and combined flow measurement of the runoff into the sea. This method was verified, and good results were achieved. Zhu Qiaoyun et al. (2008) [17] used a fixed-site Acoustic Doppler Current Profiler (ADCP) to realize real-time monitoring of cross-section flow at Xuliujing Hydrological Station, based on the principle of flow measurement, using the representative line method. Looking at medium and small rivers and tidal streams, Wei Lixin et al. (2019) [18] took the Nanjing Hydrological Experimental Station in the tidal reach of the lower Yangtze River as an example, established a multiple linear regression model between the average flow velocity and the level of the measured section, and determined the flow velocity of the ADCP index at a fixed site.

The discharge of the JLR Estuary into the sea is a key parameter that affects the flux of pollutants in the XMB, and it is an important reference basis for the Xiamen Municipal Government to conduct marine environmental pollution control. Before 2018, the pollutant fluxes into the sea were estimated, based on monthly monitoring data measured at the Beixi and Xixi gauge stations. The contribution of pollution produced in the estuary area and the migration, transformation, and removal process of pollutants during the period were never considered. Therefore, the calculated pollutants in the JLR Basin pass into the sea. This amount reflects a serious error. Wang Weiping (2006) and Zhou Zengrong (2021) [19,20] also used annual runoff to estimate the pollutant flux of the JLR, which could not be accurate to the hour or day time period. To address this problem, in this study, we constructed a model for estimating the runoff of the JLR into the sea. We used the simultaneous observation of water quality and water volume based on fixed-site buoys to estimate the load flux. This study used refined flow data to improve the accuracy of the radial flux estimation results and, thus, provided more scientific and reliable basic data for load flux estimation research.

## 2. Materials and Methods

### 2.1. Study Area

The JLR is the second-largest river in Fujian, with an average annual runoff of 149 × 10^8^ m^3^ and an average annual suspended load and sediment content of 0.210 kg/m^3^. The coordinates of the basin range are between 116°46′55″–118°02′17″ E and 24°23′53″–25°53′38″ N. The basin area is 1.47 × 10^4^ km^2^, accounting for about 12% of the land area of Fujian Province. Its main stream is composed of Beixi, Xixi, and Nanxi. The Beixi drainage area is 9803 km^2^ and the Xixi drainage area is 3964 km^2^. Beixi and Xixi converge in Zhangzhou. Nanxi flows into the Fugong, and finally flows into XMB. XMB mainly includes the west sea area, the estuary of the JLR, the southern sea area, the eastern sea area, and the Tongan Bay area.

**Figure 1:**
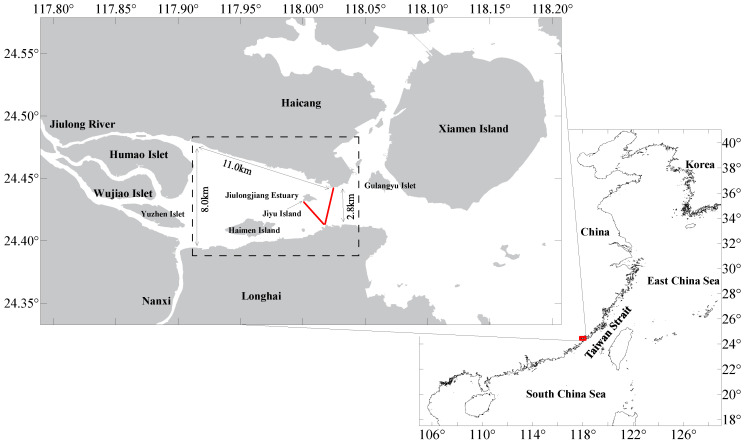
JLR–XMB in the study area.

The study area includes the estuary of the JLR, the southern part of the western sea area, and part of the southern sea area. Figure 1 shows the geographical location map of the JLR–XMB. The red line indicates the route of ADCP navigation observation. The XMB estuary of the JLR is about 11.0 km from Humao Islet to Haicang Songyu port area from east to west, and the north–south width gradually narrows from about 8.0 km to about 2.8 km between the Songyu port area and Zhangzhou port area. The research on the flow volume of the JLR into the sea adopted the method of representative site combined with cross-sectional navigation observation. The monitoring section was selected in the vicinity of about 2.8 km between the Songyu port area and the Zhangzhou port area. The Jiyu Island, which is 1 km upstream of this section, divides the river into two narrow and deep river sections under natural conditions. It is also affected by the reciprocating motion of tidal current and runoff. Therefore, it offers good representativeness and was used as the monitoring and control section of the runoff into the sea. The waters on both sides of Jiyu Island are empty, without artificial structures, sea surface breeding facilities, or obvious reef distribution, which was conducive to the observation of ships on the sea.

### 2.2. Navigation Observation

**Figure 2:**
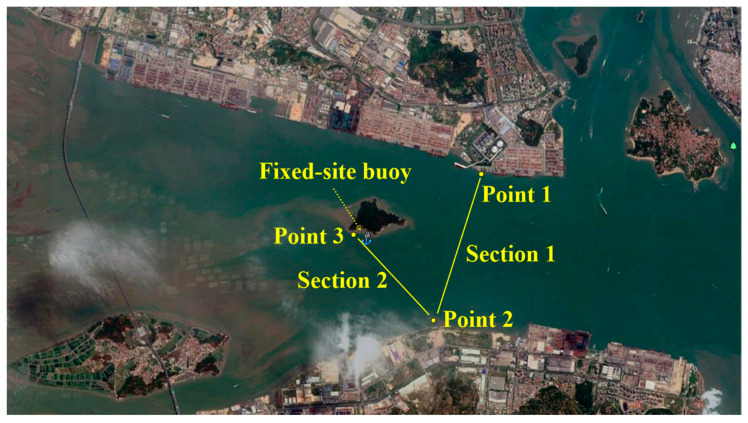
ADCP navigation section and fixed-site station at Jiulongjiang Estuary.

In this study, we carried out observations and collected the ADCP section navigation observation data for two sections along the JLR Estuary, see Appendix A data. Specifically, data included hourly observations for a tidal cycle (13 consecutive hours) in the spring and neap tides during the normal, wet, and dry seasons. The specific time for the normal water period was 23 April 2020 (lunar calendar on the first day of April), and 30 April 2020 (Lunar calendar on the eighth day of April); for the wet period it was 21 July 2020 (Lunar calendar on the first day of June), and 28 July 2020 (lunar calendar on the eighth day of June); and for the dry season it was 15 November 2020 (lunar calendar on the first day of October), and 22 November 2020 (lunar calendar on the eighth day of October). The schematic diagram of the location of the two navigation sections is shown in Figure 2. Section 1 was the route from point 1 (24.44156° N, 118.02496° E), near Haicang Port, to point 2 (24.41638° N, 118.01753° E), near Zhangzhou Port. The length was about 2.8 km. Section 2 was from point 2 of Zhangzhou Port to point 3 near Jiyu Island (24.43093° N, 118.00107° E). The length was about 2.0 km.

We took navigation observations once an hour, and it took about 25 min to complete the observation of the two cross-sections each time. The observation time started at 6 o’clock on the observation day. First, we observed and recorded the observation data from point 1 to point 2, completed the observation of section 1 and recorded a set of observation data files of section 1. Then the ship continued to sail from point 2 to point 3, and we recorded the observation data file of section 2. After a break until the next hour, the observation started from point 3 to point 2, and the observation data file for section 2 was recorded. Then, the measuring ship started from point 2 to point 1 and recorded the observation data file for section 1. The ship went back and forth until the end of the same day’s observation work at 18:00. The start time and end time of the hourly navigation observations were generally between 15 min before the hour and 10 min after the hour.

The instrument used for cross-section navigation observation was the WHS600 ADCP, by RDI Company (Spartanburg, SC, USA). During the measurement, the ADCP was installed on the side of the ship, the sensor probe was placed down about 50 cm below the water surface, and the probe was connected to a notebook computer through the RS232 serial port. We then ran the winRiver software, which is a special software for ADCP navigation flow observation, developed by RDI Company of the United States.

ADCP receives and processes the echo signal from the river bottom or the seabed to track the movement of the seabed through “bottom tracking.” As there is no moving suspense on the seabed, the speed measured by “bottom tracking” is the ship speed. The speed measured by ADCP by tracking the movement of suspended particulate matter in the water body is the speed of water flow relative to the ADCP instrument. WinRiver software automatically deducts the ship speed measured by “bottom tracking,” that is, the speed of ADCP’s own movement, which is the absolute speed of water flow movement.

**Figure 3:**
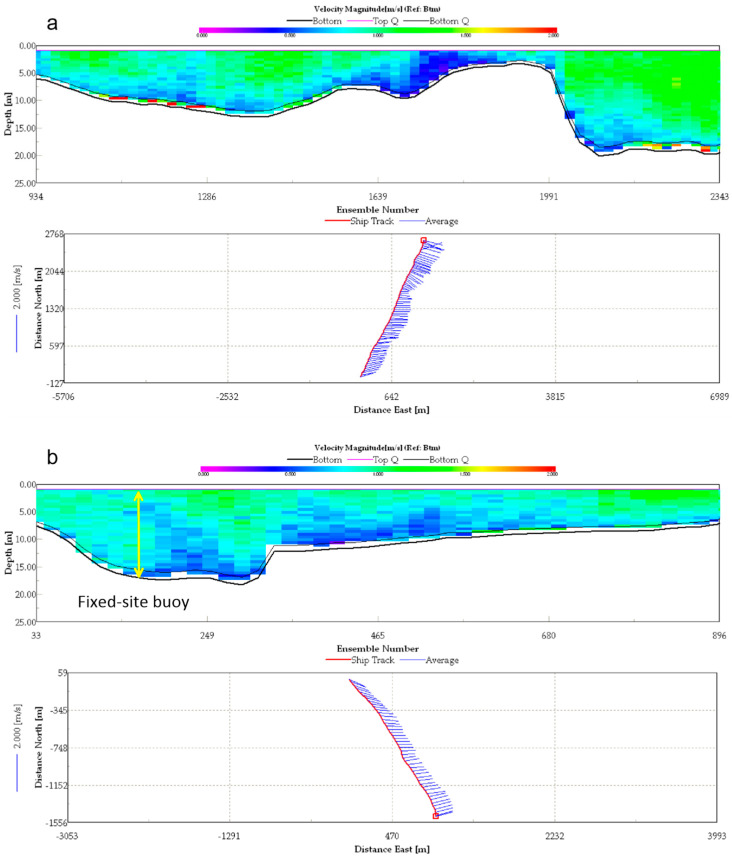
Section 1 and Section 2 screenshot of winRiver software in the wet season spring tide (21 July 2020 15:00). (**a**) Section 1 flow velocity and ship track from left Zhangzhou port to right Haicang port (**b**) Section 2 flow velocity and ship track from left Jiyu Island to right Zhangzhou port.

The stratification set during ADCP navigation observation was 0.5 m, the time interval of each data ping was 0.65 s, and the effective rate of all data was 98%. Figure 3 shows screenshots of winRiver software operation during the field survey. The cross-sectional shapes of these two sections are shown in the figure. In Figure 3a, point 1 of the Haicang port area is depicted on the right side of section 1, and the water depth of the port pool was about 20 m; point 2 of the shore near Zhangzhou Port is depicted on the left, and the water depth was about 5 m; and the shallowest water depth in the middle section was about 3 m, which was the shoal on the east side of Jiyu Island. In Figure 3b, point 2 near Zhangzhou Port is depicted on the right, and point 3 near the Bank of Jiyu Island is depicted on the left, with a water depth of about 7 m.

The ADCP flow calculation method was first proposed by Christensen and Herrick (1982) [21] and Simpson and Oltmann (1993) [22]. They used the vector cross-product of unit velocity and ship velocity to deduce the calculation formula. In this study, the vector cross-product and the appropriate depth average velocity of micro section were used to deduce the formula. ADCP uses the following formula to calculate the flow:

(1) $$Q=\underset{S}{\iint }u\times \xi ds$$

where *Q* is flow rate, *S* is the cross-sectional area of the river mouth, *u* is velocity vector at a certain point on the river cross-section, *ξ* is unit normal vector on the track of the observation ship, and *ds* is the micro-element area on the river cross-section.

*ds* is determined by the following formula:

(2) $$ds=\left|V_{b}\right|dz\times dt$$

where *dz* is length of vertical micro-element, *dt* is time micro-element, *V_b_* is vessel speed vector, |*V_b_*| is working boat speed (along track), *z* is vertical coordinate, *z* is 0 is the river bottom, *z* is H is the water surface (H is water depth).

|*V_b_*| is determined by the following formula:

(3) $$\left|V_{b}\right|=\sqrt{V_{bx}^{2}+V_{by}^{2}}$$

where *V_bx_* and *V_by_* are the ship’s speed in the *x* and *y* directions, and (*x*, *y*) is the relative position of the ADCP sailing on the horizontal plane, *x* is in east–west direction, *y* is in north–south direction.

The above flow Formulae (1)–(3) can be rewritten as follows:

(4) $$Q=\int_{0}^{T}\left[\int_{0}^{H}u\times dz\right]\;\times \xi \;|V_{b}|\times dt\;=\int_{0}^{T}\int_{0}^{H}\left(u\times V_{b}\right)\;\times k\times dz\;dt$$

where *T* is cross-section navigation time, and *k* is vertical unit vector.

**Figure 4:**
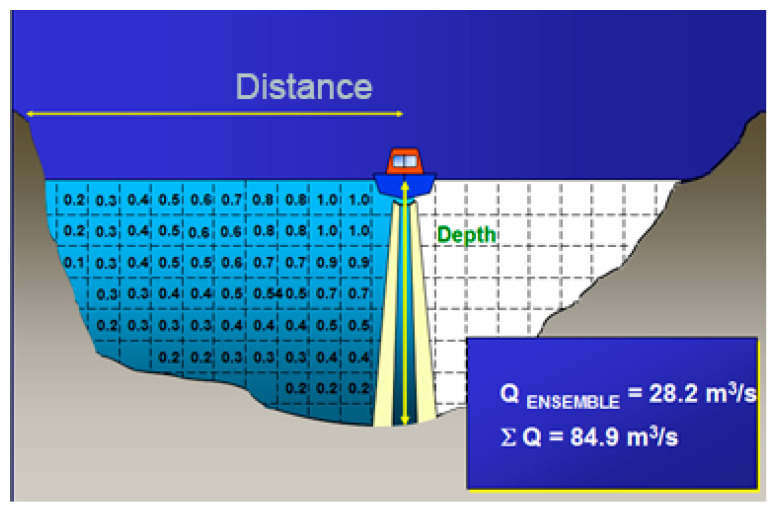
Schematic diagram of the principle of ADCP flying aerial flow measurement.

A schematic diagram depicting the principle of ADCP navigation flow observation is shown in Figure 4. In the figure, Q_ENSEMBLE_ is section flow of acoustic wave coverage area at the observation time, ∑Q is sum of flow through the section for flow measurement, The numerical marks on the left section grid are the stratified velocities of each section measured by ADCP flow measurement, and the section without digital marks on the right side is where ADCP did not measure the section. Distance is the straight-line distance of ADCP measured section. The boat in the picture is sailing from left to right. The boat pattern is just a sign, not the direction of sailing. The light beam represents the direction of sound wave sent by ADCP, usually within 20° from the vertical direction. There is no sound wave in the center, and the green color represents the color of the water body.

### 2.3. Buoy Data Collection

In this study, we also monitored and collected fixed-site buoy ADCP online, in the form of hourly current profile velocity and flow direction data. The schematic diagram of the fixed-site buoy ADCP station is shown in Figure 2. The latitude and longitude were 24°25.700′ N, 118°00.516′ E, located near the deep groove of the waterway in the south of Jiyu Island, about 300 m away from the bank of Jiyu Island. The actual water depth was between 12 m and 18 m. The relative position of this section is shown in Figure 3.

The fixed-site buoy was the water quality environmental monitoring buoy of Xiamen Environmental Monitoring Station, which was loaded with Norway AANDERAA Seaguard II-DCP. The main technical indicators were as follows: velocity measurement range 0–3 m/s, accuracy: 0.3 cm/s or ±1.5% FS, resolution 0.2 cm/s. The layer of ADCP current profile was 1 m, the working frequency was 600 kHz, the current measurement accuracy was ±0.25% of the observed value, and the current measurement resolution was 1 mm/s. The time range of the data obtained was from April 2020, to March 2021. In this study, we mainly took data from the same period as the navigation observation for comparative analysis.

**Figure 5:**
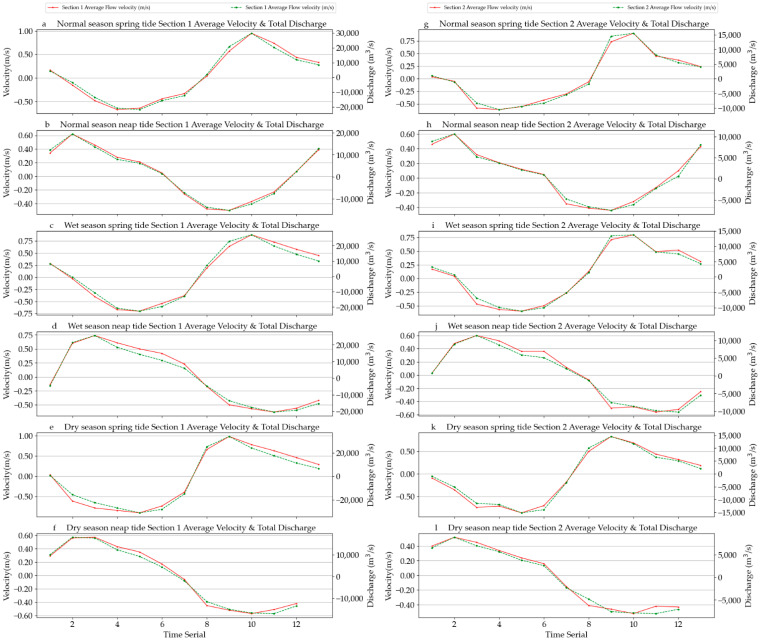
Process curve of the average velocity and total discharge of two sections at the spring tide and neap tide during the normal season, wet season, and dry season. (**a**) Normal season spring tide average velocity & total discharge of section 1, (**b**) Normal season neap tide average velocity & total discharge of section 1, (**c**) Wet season spring tide average velocity & total discharge of setion 1, (**d**) Wet season neap tide average velocity & total discharge of section 1, (**e**) Dry season spring tide average velocity & total discharge of section 1, (**f**) Dry season neap tide average velocity & total discharege of section 1, (**g**) Normal season spring tide average velocity & total discharge of section 2, (**h**) Normal season neap tide average velocity & total discharge of section 2, (**i**) Wet season spring tide average velocity & total discharge of setion 2, (**j**) Wet season neap tide average velocity & total discharge of section 2, (**k**) Dry season spring tide average velocity & total discharge of section 2, (**l**) Dry season neap tide average velocity & total discharege of section 2.

In 2020, there were six navigation times during which we observed two sections throughout the normal water period, wet season, dry season, spring tide, and neap tide. During the observation period, winRiver software statistically output the observation report, including the main parameters, average velocity, flow direction, total cross-section flow, and cross-section area. In addition, we collected the hourly tide-level data of the Gulangyu marine station, which was 5 km away from the east side of Jiyu Island (see Appendix A Table A1 for details). Since the flux has a direction, for the convenience of counting the runoff flow in this section, we took the direction flowing into the ocean as positive and the reverse as negative. Therefore, for the average velocity and the discharge data of the section given in the table, we expressed the falling tide as positive. The discharge flowed into the sea through the section downstream, the rising tide was expressed as negative, and the discharge was the sea water flowing back through the section upstream. Figure 5 provides the process curve of the section’s average velocity and the section’s discharge during each observation period.

## 3. Results

### 3.1. Analysis of ADCP Section Navigation Data

The statistics of section navigation observation data in spring tide and neap tide are shown in Table 1 and Table 2.

According to the data’s statistics, the average velocity of the section could be classified according to the spring and neap tides. The maximum value of all profile average velocity in the two sections, observed three times in the wet, normal, and dry seasons, during the spring tide, was 0.60–0.98 m/s (average 0.81 m/s), and the average value of all profile average velocity in the two sections was 0.37–0.71 m/s (average 0.49 m/s). During the neap tide, the maximum value of all profile average velocity of the two sections observed three times, during the wet, normal, and dry seasons, was 0.44–0.74 m/s (average 0.57 m/s), and the average value of all profile average velocity was 0.28–0.52 m/s (average 0.38 m/s). The maximum value of the section profile average velocity during the spring tide was 1.42 times that during the neap tide, and the average value of the section profile average velocity was 1.29 times that during the neap tide. The velocity was affected mainly by the tidal range. The statistics of the measured tidal level data showed that the ratio of the maximum tidal range of the spring and neap tides was 1.50:1.

On the basis of the statistical results of the discharge of the section, according to the classification of the spring and neap tides, the maximum value of the discharge of section 1, during spring tide, was 21,784–34,005 m^3^/s (average 27,337 m^3^/s), and the average value was 13,855–23,268 m^3^/s (average 16,498 m^3^/s). During the neap tide, the maximum discharge of section 1 was 15,226–19,293 m^3^/s (average 19,293 m^3^/s), and the average was 9498–16,113 m^3^/s (average 12,505 m^3^/s). The maximum section discharge during the spring tide was 1.42 times that during the neap tide, and the average section discharge was 1.32 times that during the neap tide.

According to the classified statistics of the wet, normal, and dry seasons, the maximum velocity of the ebb tide in section 1, during spring, was 0.88 m/s in the wet season (July 2020), 0.95 m/s in the normal season (April 2020), and 0.98 m/s in the dry season (November 2020). The maximum velocity ratio of the spring tide, section 1, in the wet, normal, and dry seasons was 1:1.08:1.11. The tidal range ratio in the corresponding period was 1:0.87:1.19.

According to the classified statistics of the wet, normal, and dry seasons, the average value of the maximum velocity of the ebb tide in section 1, during the neap tide, was 0.74 m/s in the wet season (July 2020), 0.62 m/s in the normal season (April 2020), and 0.57 m/s in the dry season (November 2020). The maximum velocity ratio of spring tide, section 1, in wet, normal, and dry seasons, was 1:0.84:0.77. The tidal range ratio in the corresponding period was 1:0.58:0.81.

Similarly, the ratio of the average value of the maximum velocity of the spring and ebb tides of section 2 was 1:13:1.04, and the ratio of the average value of the maximum velocity of the neap and ebb tides of section 2 was 1:1:0.87. The ratio of the maximum value of the discharge of spring tide, section 1, was 1:1.10:1.26, and the ratio of the maximum value of the discharge of neap tide, section 1. was 1:0.76:0.70. The ratio of the maximum value of the discharge of spring tide, section 2, was 1:1.13:1.10, and the ratio of the maximum value of the discharge of neap tide, section 2, was 1:0.93:0.78.

The statistical data showed that the characteristics of wet, normal, and dry seasons during the neap tide conformed to the general law that the maximum velocity and discharge of the section were greater in the wet season than in the normal season, and greater than in the dry season. No such law applied during spring tide, and the maximum velocity and discharge of the section were larger in the dry season because of the larger tidal range, indicating that the maximum velocity and discharge of the section were affected mainly by tide, rather than by season.

### 3.2. Analysis of ADCP Current Profile Data of Fixed-Site Buoy

**Figure 6:**
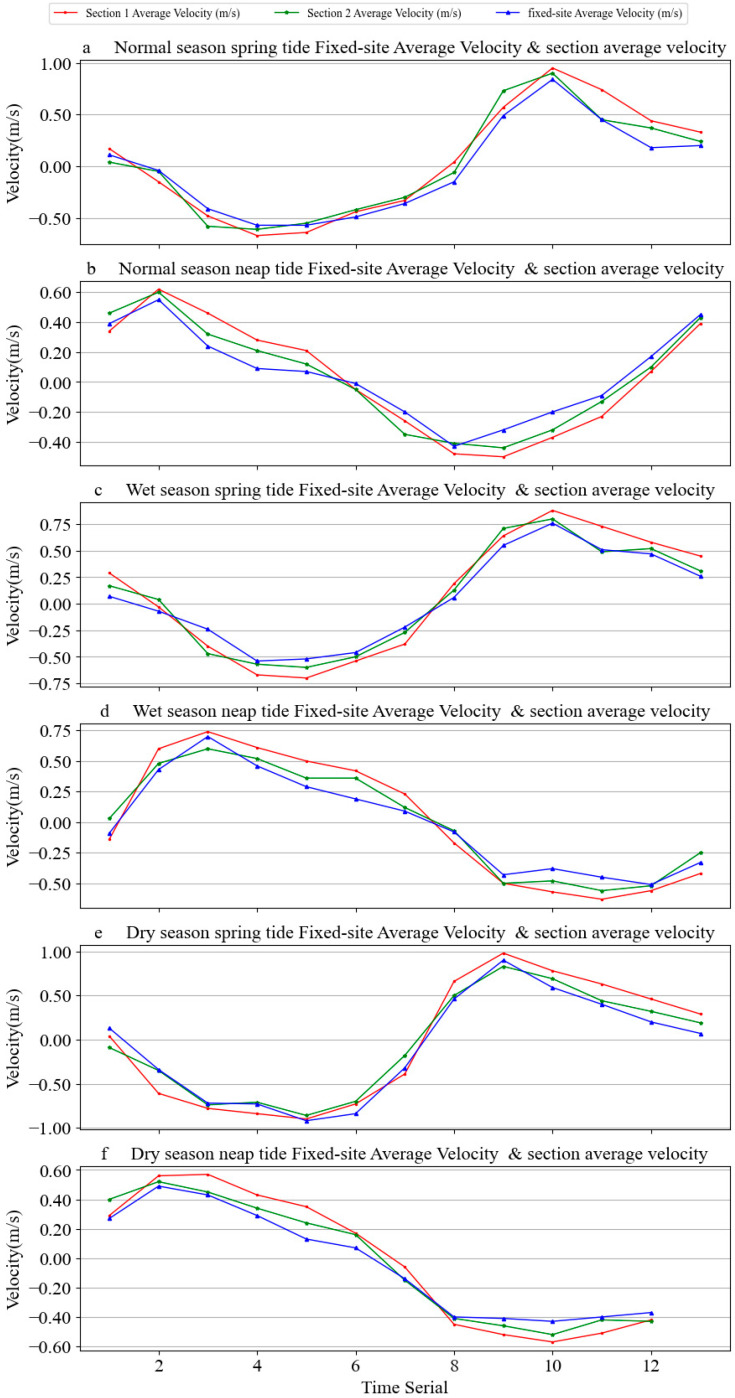
Process curve of fixed-site buoy vertical average velocity and section average velocity. (**a**) Normal season spring tide average velocity & section average velocity of the fixed-site, (**b**) Normal season neap tide average velocity & section average velocity of the fixed-site, (**c**) Wet season spring tide average velocity & section average velocity of the fixed-site, (**d**) Wet season neap tide average velocity & section average velocity of the fixed-site, (**e**) Dry season spring tide average velocity & section average velocity of the fixed-site, (**f**) Dry season neap tide average velocity & section average velocity of the fixed-site.

Figure 6 shows the process curve between the vertical average velocity of ADCP current and the average velocity of two sections. The process curve indicates that the vertical average velocity of the current of the fixed-site buoy was consistent with the magnitude and change trend of the average velocity of the two sections, and the relevant statistical data and laws were consistent.

The maximum average velocity of the spring tide was 0.76–0.90 m/s (average 0.83 m/s), and the maximum vertical average velocity of the neap tide was 0.49–0.70 m/s (average 0.58 m/s). The maximum vertical average velocity of the fixed-site buoy in the spring tide was 1.43 times that in the neap tide.

The maximum velocity in the wet season was 0.76 m/s, the maximum velocity in the normal season was 0.84 m/s, and the maximum velocity in the dry season was 0.90 m/s, which also showed that the velocity was affected mainly by the tide, rather than by the season.

## 4. Discussion

### 4.1. Correlation Formulae between Tide Level, Section Average Velocity, and Total Discharge

**Figure 7:**
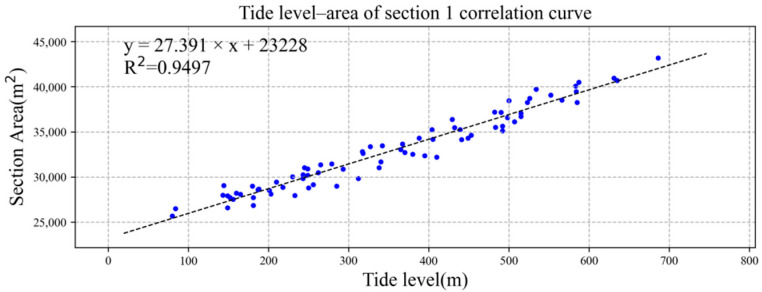
Linear correlation between tide level and section 1 area. The data of blue dots represent the relationship between tide level (x) and area of section 1 (y).

**Figure 8:**
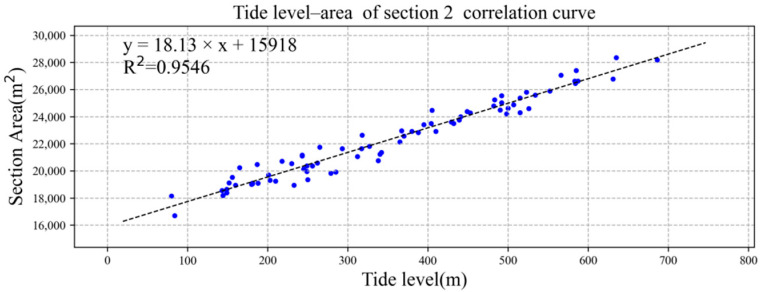
Linear correlation between tide level and section 2 area. The data of blue dots represent the relationship between tide level (x) and area of section 2 (y).

We drew the tide level and the section area of section 1 and section 2 into an XY point aggregation diagram in Microsoft Excel software and added the trend line, as shown in Figure 7 and Figure 8. Both had good linear correlation, and the correlation coefficient R^2^ was greater than 0.90. The correlation formulae and correlation coefficiente between tide level and section area of section 1 and section 2 were as follows:

S_1_ = 27.391TL + 23228, R^2^ = 0.9497, (5)

S_2_ = 18.130TL + 15918, R^2^ = 0.9546, (6)

where S_1_ is the cross-sectional area of section 1 (m^2^), S_2_ is the cross-sectional area of section 2 (m^2^), and TL is the tide level (cm).

**Figure 9:**
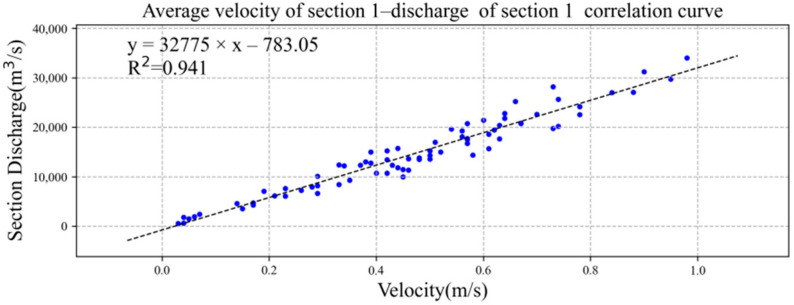
Linear correlation between average velocity of section 1 and total discharge of section. The data of blue dots represent the relationship between average velocity of section 1 (x) and total discharge of section 1 (y).

**Figure 10:**
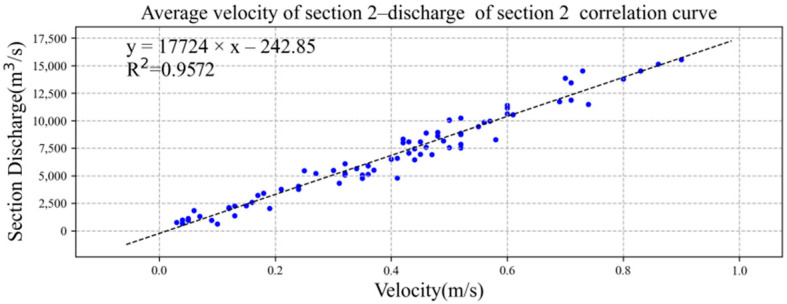
Linear correlation between average velocity of section 2 and total discharge of section. The data of blue dots represent the relationship between average velocity of section 2 (x) and total discharge of section 2 (y).

We drew the average flow velocity and total discharge of sections 1 and 2 into the XY point aggregation diagram in Microsoft Excel software and added the trend line, as shown in Figure 9 and Figure 10. Both had good linear correlation, and the correlation coefficient R^2^ was greater than 0.90. The correlation formulae and coefficients between the average velocity of section 1 and section 2 and the discharge of section were as follows:

*Q*_1_ = 32,775*u*_1_ − 783.05, R^2^ = 0.9410, (7)

*Q*_2_ = 17,724*u*_2_ − 242.85, R^2^ = 0.9572, (8)

where *Q*_1_ is the total discharge (m^3^/s) of section 1, *Q*_2_ is the total discharge (m^3^/s) of section 2, *u*_1_ is the average flow speed (m/s) of section 1, and *u*_2_ is the average flow speed (m/s) of section 2.

### 4.2. Correlation Formula between the Average Velocity of the Fixed-Site Buoy and the Average Velocity of the Section

**Figure 11:**
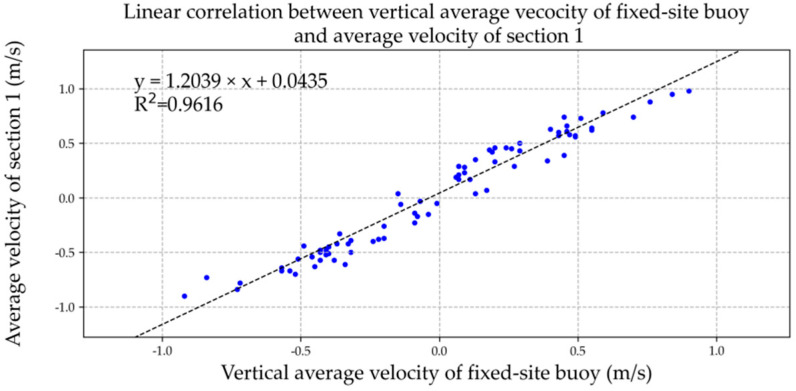
Linear correlation between vertical average current of fixed-site buoy and average velocity of section 1. The data of blue dots represent the relationship between vertical average velocity of the fixed-site buoy (x) and average velocity of section 1 (y).

**Figure 12:**
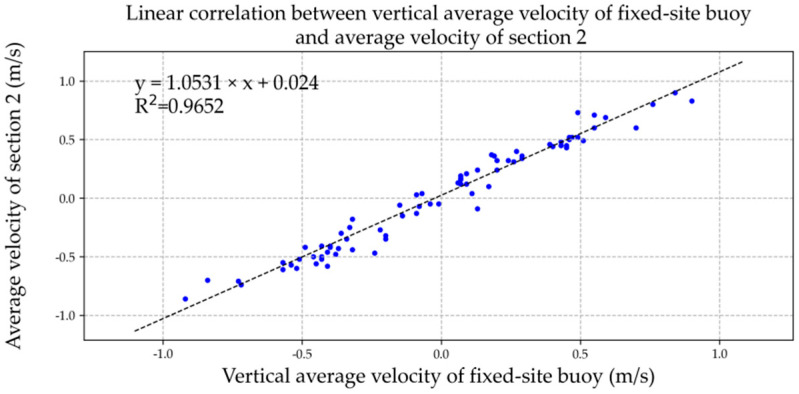
Linear correlation between vertical average current of fixed-site buoy and average velocity of section 2. The data of blue dots represent the relationship between vertical average velocity of the fixed-site buoy (x) and average velocity of section 2 (y).

We drew the vertical average velocity of the fixed-site buoy and the average velocity of the two sections in Microsoft Excel software and added the XY point aggregation diagram with the trend line for correlation analysis, as shown in Figure 11 and Figure 12. The relevant formulae and coefficients were as follows:

*u*_1_ = 1.2039*V_b_* + 0.0435, R^2^ = 0.9616, (9)

*u*_2_ = 1.0531*V_b_* + 0.0240, R^2^ = 0.9652, (10)

where *u*_1_ is section 1 average flow speed (m/s), *u*_2_ is section 2 average flow speed (m/s), and *V_b_* is the vertical average velocity of ADCP current profile of fixed-site buoy (m/s).

### 4.3. Correlation Formula between Fixed-Site Vertical Average Velocity and Section Discharge

Method 1:

Using the formula “total discharge of section = average velocity of section × section area”, we replaced relevant Formulae (9) and (10) to calculate the average velocity of both sections and the measured tide level with relevant Formulae (5) and (6) to obtain the tide level and section area of section 1 and section 2, as follows:

*Q*_1_ = (1.2039*V_b_* + 0.0435) × (27.391TL + 23228), (11)

*Q*_2_ = (1.0531*V_b_* + 0.0240) × (18.130TL + 15918), (12)

where *Q*_1_ is section 1 total discharge (m^3^/s), *Q*_2_ is section 2 total discharge (m^3^/s), *V_b_* is the vertical average velocity of the current of the fixed-site buoy (m/s), and TL is the tide level (cm).

Method 2:

By substituting correlation Formulae (9) and (10) between the average velocity of the two sections and the velocity of the buoy ADCP current profile with correlation Formulae (7) and (8) between the average velocity of section 1 and section 2 and the total discharge of section, the following could be calculated:

*Q*_1_ = 32,775 (1.2039*V_b_* + 0.0435) − 783.05, (13)

*Q*_2_ = 17,724 (1.0531*V_b_* + 0.0240) − 242.85, (14)

where *Q*_1_ is section 1 total discharge (m^3^/s), *Q*_2_ is section 2 total discharge (m^3^/s), and *V_b_* is the vertical average velocity of the current of the fixed-site buoy (m/s).

## 5. Conclusions

### 5.1. Error Analysis

**Figure 13:**
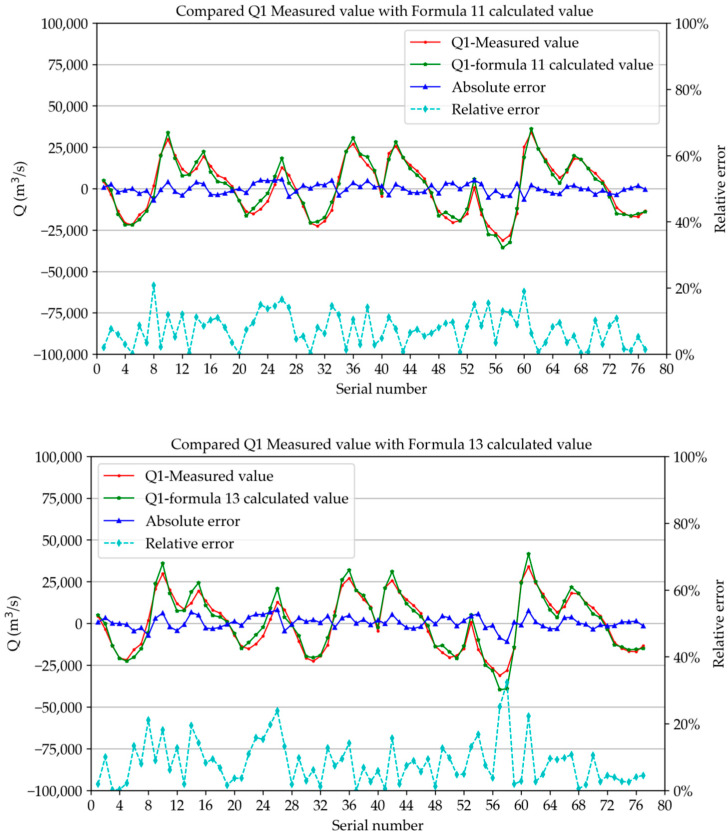
Comparison between *Q*_1_ measured value and calculated value of formula.

**Figure 14:**
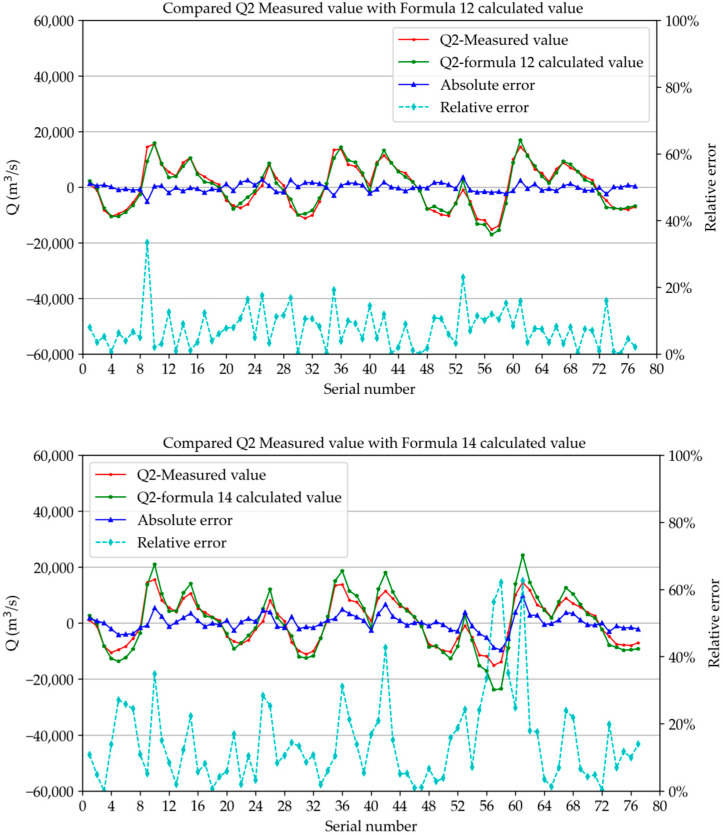
Comparison between *Q*_2_ measured value and calculated value of formula.

In this study, we calculated the discharge of the two sections during the observation period using the correlation formula between the fixed-site vertical average velocity and the section discharge. We compared the measured discharge during six observation periods with the calculated discharge, and the relative deviation percentage value was given, as shown in Figure 13 and Figure 14. The figures show that the measured value of *Q*_1_ (the discharge of section 1) was very close to the calculated value of relevant Formulae (11) and (13), and the measured value *Q*_2_ (the discharge of section 2) was very close to the calculated value of relevant Formulae (12) and (14). As shown in the figures, the overlap of the two curves was very good. The average values of relative error were section 1 (Formula (11)): 7.4%, section 1 (Formula (12)): 8.4%, section 2 (Formula (12)): 7.6%, and section 2 (Formula (14)): 14.9%. The mean square deviations were 5.7%, 6.3%, 5.7%, and 8.0%, respectively. It can be seen that Formulae (11) and (12) in method 1 had fewer errors and higher accuracy and could be used as the recommended formula for subsequent research and calculation.

### 5.2. Ideas for Improving Observation Method

In this project, to study the runoff and circulation of the JLR into the sea, we adopted the method of one representative site in the south of Jiyu Island, combined with the navigation observation of two sections. As this representative site was located in the mainstream area of the waterway in the south of Jiyu Island and had good representativeness, the correlation between the representative vertical average velocity and the discharge on the section was good. Thus, the correlation Formulae (11)–(14) established in this paper can be used. The discharge of the section was calculated according to the vertical average velocity of the buoy observed in real time, and then the real-time pollutant flux of the section was calculated along with the pollutant concentration monitoring data.

The tide current reverse period between the north and south sides of Jiyu Island, was different near the high-level and low-level tides. The current direction on the north side of Jiyu Island was still the direction of rising tide, and the current direction on south side of Jiyu Island turned to the direction of falling tide. To improve the calculation accuracy of the discharge of this section, we set a representative observation site on the north side of Jiyu Island for profile current observation. The Haicang port area on the north side of Jiyu Island is a large-scale ship berthing and turning water area, and dredging is often required. Therefore, it was not suitable to set a sea buoy or seabed-based ADCP in the main channel area of the port area to conduct long-term real-time online detection. We did set H-ADCP at an appropriate position along the shore of the wharf berth for horizontal section observation [16,23,24,25]; that is, the representative layer flow measurement method. The combined flow measurement method, formed by one representative observation site and one representative layer flow measurement method, combined with the navigation observations of these two sections, further improved the accuracy of real-time online monitoring of section discharge.

## Figures and Tables

**Table 1:** Statistics of Section Navigation Observation Data in Spring Tide.

Season	Spring Tide	Section 1		Section 2	
		Velocity m/s	Discharge m^3^/s	Velocity m/s	Discharge m^3^/s
Normal season	Average in ebb tide	0.46	13,855	0.46	8120
	Average in rising tide	0.45	14,628	0.37	6444
	Maximum in ebb tide	0.95	29,725	0.90	15,548
	Maximum in rinsing tide	0.67	21,784	0.61	10,558
Wet season	Average in ebb tide	0.54	15,598	0.40	6560
	Average in rising tide	0.45	14,550	0.48	8661
	Maximum in ebb tide	0.88	27,058	0.80	13,771
	Maximum in rinsing tide	0.70	22,635	0.60	11,164
Dry season	Average in ebb tide	0.55	17,092	0.50	8318
	Average in rising tide	0.71	23,268	0.52	8829
	Maximum in ebb tide	0.98	34,005	0.83	14,512
	Maximum in rinsing tide	0.90	31,214	0.86	15,121

**Table 2:** Statistics of Section Navigation Observation Data in Neap Tide.

Season	Neap Tide	Section 1		Section 2	
		Velocity m/s	Discharge m^3^/s	Velocity m/s	Discharge m^3^/s
Normal season	Average in ebb tide	0.30	9498	0.29	5031
	Average in rising tide	0.37	11,239	0.33	5435
	Maximum in ebb tide	0.62	19,447	0.60	10,616
	Maximum in rinsing tide	0.50	15,226	0.44	7456
Wet season	Average in ebb tide	0.52	16,113	0.35	6133
	Average in rising tide	0.43	13,612	0.40	7176
	Maximum in ebb tide	0.74	25,650	0.60	11,404
	Maximum in rinsing tide	0.63	20,401	0.56	10,254
Dry season	Average in ebb tide	0.40	11,985	0.35	5722
	Average in rising tide	0.42	12,585	0.40	6267
	Maximum in ebb tide	0.57	18,080	0.52	8845
	Maximum in rinsing tide	0.57	16,954	0.52	8001

## Data Availability

The data that support the findings of this study are available from the corresponding author, upon reasonable request.

## Appendix A

**Table A1:** Section navigation observation data report.

Date	Section 1						Section 2						Tide Level m
	Start Time	Finish Time	Average Velocity m/s	Direction °	Discharge m^3^/s	Area m^2^	Start Time	Finish Time	Average Velocity m/s	Direction °	Discharge m^3^/s	Area m^2^	
**23 April 2020** **Normal season spring tide**	6:12	6:28	0.17	121	4300	26,866	6:28	6:40	0.04	34	993	19,053	181
	7:01	7:16	−0.15	296	−3544	27,715	6:49	7:01	−0.05	255	−1112	19,083	181
	7:45	8:00	−0.48	281	−13,504	30,261	8:00	8:12	−0.58	281	−8289	21,100	243
	8:59	9:14	−0.67	290	−20,816	31,693	8:46	8:59	−0.61	275	−10,558	21,235	340
	9:45	10:00	−0.64	284	−21,784	35,486	10:00	10:12	−0.55	277	−9472	23,496	432
	11:00	11:15	−0.44	285	−15,736	36,127	10:47	11:00	−0.42	274	−8341	24,879	507
	11:46	12:01	−0.33	274	−12,383	38,534	12:01	12:12	−0.3	282	−5491	27,051	566
	12:59	13:16	0.04	113	1794	38,281	12:47	12:59	−0.06	227	−1848	27,396	585
	13:47	14:02	0.57	112	20,720	38,257	14:02	14:17	0.73	96	14,519	25,814	523
	14:57	15:13	0.95	105	29,725	32,372	14:46	14:57	0.9	98	15,548	23,399	395
	15:46	16:02	0.74	114	20,175	31,467	16:03	16:15	0.45	88	8098	19,817	279
	16:59	17:15	0.44	100	11,842	28,110	16:46	16:58	0.37	98	5502	19,307	203
	17:45	18:02	0.33	120	8427	27,910	18:02	18:15	0.24	88	4057	18,636	149
**30 April 2020** **Normal season neap tide**	5:38	5:54	0.34	113	12,214	35,624	5:54	6:07	0.46	96	8879	25,018	492
	6:57	7:12	0.62	103	19,447	32,212	6:47	6:57	0.6	96	10,616	22,917	410
	7:47	8:03	0.46	108	13,617	31,044	8:03	8:16	0.32	96	5216	20,759	338
	8:56	9:10	0.28	111	7934	28,990	8:45	8:56	0.21	86	3779	19,903	285
	9:47	10:02	0.21	104	6153	28,807	10:02	10:14	0.12	92	2107	19,353	250
	10:54	11:08	0.05	102	1458	27,952	10:43	10:54	0.05	67	948	18,945	233
	11:48	12:03	−0.26	279	−7219	29,167	12:03	12:14	−0.35	282	−4759	20,363	256
	12:56	13:10	−0.48	284	−13,809	29,848	12:44	12:56	−0.41	277	−6603	21,063	312
	13:47	14:03	−0.5	283	−15,226	32,525	14:03	14:14	−0.44	279	−7456	22,912	380
	14:58	15:12	−0.37	291	−12,350	34,144	14:45	14:58	−0.32	273	−6097	23,982	441
	15:47	16:02	−0.23	270	−7590	35,522	16:02	16:13	−0.13	282	−2260	25,243	483
	16:55	17:10	0.07	69	2395	35,125	16:42	16:55	0.1	126	622	25,551	492
	17:47	18:02	0.39	112	12,769	34,623	18:02	18:15	0.43	94	8079	24,261	453
**21 July 2020** **Wet season spring tide**	5:42	5:57	0.29	119	8201	29,455	5:57	6:07	0.17	77	3231	19,231	210
	6:55	7:11	−0.03	339	−555	28,517	6:45	6:55	0.04	73	679	19,694	201
	7:46	8:03	−0.40	275	−10,700	30,198	8:03	8:13	−0.47	278	−6920	20,357	249
	8:54	9:11	−0.67	287	−20,755	33,452	8:44	8:54	−0.57	271	−9966	21,353	342
	9:45	10:00	−0.70	280	−22,635	35,266	10:00	10:10	−0.6	277	−11,164	23,755	439
	10:54	11:11	−0.54	284	−19,631	38,711	10:44	10:54	−0.5	271	−10,044	24,602	526
	11:43	11:58	−0.38	272	−13,026	39,453	11:58	12:08	−0.27	282	−5209	26,446	584
	12:54	13:10	0.19	102	7050	40,078	12:44	12:54	0.13	124	1363	26,634	583
	13:43	13:59	0.64	107	22,805	38,458	13:59	14:11	0.71	93	13,434	24,626	500
	14:53	15:08	0.88	102	27,058	33,666	14:43	14:53	0.8	95	13,771	22,960	367
	15:44	16:03	0.73	115	19,765	30,911	16:03	16:14	0.49	87	8159	19,944	249
	16:53	17:08	0.58	100	14,348	27,745	16:42	16:53	0.52	94	7525	19,102	152
	17:43	18:01	0.45	114	9957	25,688	18:01	18:14	0.31	93	4317	18,135	80
**28 July 2020** **Wet season neap tide**	5:44	6:00	−0.14	260	−4559	40,511	6:00	6:11	0.03	48	756	26,627	587
	6:53	7:09	0.60	98	21,418	39,709	6:44	6:53	0.48	99	8954	25,587	534
	7:48	8:06	0.74	110	25,650	36,376	8:06	8:18	0.6	90	11,404	23,592	429
	8:53	9:09	0.61	102	18,565	33,386	8:44	8:53	0.52	94	8740	21,807	327
	9:43	9:59	0.50	108	14,305	31,031	10:00	10:10	0.36	88	5911	20,120	245
	10:54	11:09	0.42	94	10,688	28,993	10:45	10:54	0.36	98	5122	18,993	180
	11:44	12:02	0.23	114	6053	27,994	12:02	12:12	0.12	74	2045	18,555	143
	12:55	13:10	−0.17	295	−4676	28,201	12:44	12:54	−0.07	255	−1306	18,942	160
	13:44	14:00	−0.50	276	−13,586	30,035	14:00	14:09	−0.5	279	−7566	20,539	230
	14:55	15:12	−0.57	287	−17,538	32,824	14:43	14:55	−0.48	270	−8603	21,628	317
	15:44	16:00	−0.63	279	−20,401	35,247	16:00	16:09	−0.56	277	−9859	23,501	404
	16:54	17:10	−0.56	283	−19,283	37,173	16:43	16:54	−0.52	270	−10,254	24,497	490
	17:43	17:58	−0.42	280	−15,238	39,064	17:58	18:08	−0.25	262	−5468	25,881	552
**15 November 2020** **Dry season spring tide**	5:58	6:17	0.04	147	645	26,484	6:17	6:25	−0.09	283	−964	16,694	84
	7:02	7:19	−0.61	286	−15,670	29,062	6:52	7:02	−0.35	276	−5082	18,188	144
	7:51	8:07	−0.78	283	−22,523	31,350	8:07	8:17	−0.74	275	−11,472	21,751	265
	9:01	9:18	−0.84	284	−26,977	34,317	8:51	9:01	−0.71	274	−11,871	22,836	388
	9:45	10:00	−0.9	282	−31,214	37,081	10:00	10:08	−0.86	273	−15,121	24,295	515
	10:52	11:10	−0.73	281	−28,212	40,963	10:41	10:51	−0.7	271	−13,874	26,775	631
	11:43	11:57	−0.39	276	−15,009	43,192	11:58	12:07	−0.18	274	−3422	28,176	686
	12:54	13:09	0.66	102	25,236	40,684	12:45	12:54	0.5	96	10,087	28,352	635
	13:55	14:13	0.98	107	34,005	36,569	14:13	14:24	0.83	95	14,512	24,214	498
	14:50	15:04	0.78	105	24,152	33,053	14:41	14:49	0.69	91	11,743	22,144	365
	15:43	16:00	0.63	105	17,627	30,478	16:00	16:10	0.44	96	6456	20,579	262
	16:56	17:12	0.46	110	11,342	28,666	16:46	16:56	0.32	82	5078	19,093	188
	17:41	17:59	0.29	95	6640	26,588	18:00	18:08	0.19	108	2031	18,373	149
**22 November 2020** **Dry season neap tide**	5:50	6:06	0.29	99	10,085	37,179	6:06	6:16	0.4	99	6515	24,790	482
	6:53	7:10	0.56	103	18,080	34,177	6:44	6:53	0.52	98	8845	24,464	405
	7:43	8:00	0.57	108	17,735	32,622	8:00	8:10	0.45	95	6945	22,634	318
	8:50	9:05	0.43	110	12,324	29,832	8:42	8:50	0.34	86	5664	21,154	243
	9:43	10:00	0.35	107	9265	28,620	10:00	10:09	0.24	88	3775	20,476	187
	10:51	11:06	0.17	99	4423	27,550	10:41	10:50	0.16	88	2585	19,524	156
	11:43	12:01	−0.06	294	−1947	28,086	12:01	12:10	−0.15	259	−2284	20,231	165
	12:51	13:07	−0.45	276	−11,452	28,877	12:42	12:51	−0.41	285	−4808	20,706	218
	13:43	13:59	−0.52	288	−14,988	30,864	13:59	14:08	−0.46	264	−7580	21,640	293
	14:52	15:09	−0.57	277	−16,697	32,722	14:42	14:52	−0.52	281	−7871	22,563	370
	15:43	15:59	−0.51	290	−16,954	34,316	15:59	16:08	−0.42	261	−8001	24,381	449
	16:53	17:09	−0.42	272	−13,469	36,703	16:42	16:53	−0.43	280	−7058	25,378	515
